# Hopkins criteria for residual disease assessment after definitive radiotherapy in nasopharyngeal carcinoma

**DOI:** 10.1002/cam4.2790

**Published:** 2019-12-25

**Authors:** Yang Liu, Wen Long, Guannan Wang, Yuxiang Yang, Biaoshui Liu, Wei Fan

**Affiliations:** ^1^ Department of Radiation Oncology State Key Laboratory of Oncology in South China Collaborative Innovation Center for Cancer Medicine Sun Yat‐sen University Cancer Center Guangzhou P.R. China; ^2^ Department of Nuclear Medicine State Key Laboratory of Oncology in South China Collaborative Innovation Center for Cancer Medicine Sun Yat‐sen University Cancer Center Guangzhou P.R. China

**Keywords:** Epstein‐Barr virus, nasopharyngeal carcinoma, PET/CT, prognosis, response assessment

## Abstract

**Objectives:**

Assessment of viable tumor residue after definitive radiotherapy is essential in patients with nasopharyngeal carcinoma (NPC). This study aimed to investigate the use of Hopkins criteria on positron emission tomography/computed tomography (PET/CT) for posttreatment response evaluation and whether plasma Epstein‐Barr virus (EBV) DNA could bring additional value.

**Materials and methods:**

NPC patients who underwent FDG‐PET/CT scan within 26 weeks after definitive radiotherapy were retrospectively reviewed. Residual disease was evaluated by Hopkins 5‐point score. Accuracy of Hopkins criteria before and after incorporating EBV DNA was calculated. Prognostic value for locoregional failure‐free survival (LRFFS) and disease‐free survival (DFS) was analyzed.

**Results:**

One hundred and sixteen patients were evaluated. Median follow‐up time was 28.3 months (range 3.3‐92.0 months). Residual disease was found in 19 (16.4%) patients. Overall, Hopkins criteria had high specificity (86.6%; 95% CI, 78.2%‐92.7%) and negative prognostic value (NPV) (94.4%; 95% CI, 88.7%‐97.3%), while sensitivity and positive prognostic value (PPV) was 73.7% (95% CI, 48.8%‐90.9%), 51.9% (95% CI, 37.8%‐65.6%), respectively. Posttreatment plasma EBV DNA was not predictive of residual tumor (*P* = .272). PPV and accuracy were 50.0% (95% CI, 32.1%‐67.9%) and 83.0% (95% CI, 73.8%‐90.0%) after incorporating detectable EBV DNA into the scoring system. Positive PET/CT results were significantly correlated with inferior 3‐year LRFFS (95.7% vs 79.5%, *P* = .043) and 3‐year DFS (84.6% vs 54.4%, *P* = .028).

**Conclusions:**

The Hopkins criteria demonstrated high NPV and specificity in posttreatment assessment, with the potential to be a reliable prognostic indicator for locoregional failure. Combining EBV DNA with PET/CT did not improve diagnostic accuracies. PET/CT should not be performed less than 12 weeks after treatment.

## Graphical abstract

Our study evaluated the Hopkins criteria and EBV DNA in the posttreatment assessment of nasopharyngeal carcinoma. Hopkins criteria showed high NPV and specificity. EBV DNA was not associated with residual disease, and combining EBV DNA with PET/CT did not improve diagnostic accuracies.

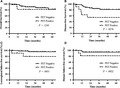

## 1 INTRODUCTION

Nasopharyngeal carcinoma (NPC) is a distinct head and neck squamous cell cancer (HSNCC) showing great geographical disparities. While it is a rare disease worldwide, NPC is endemic in Southeast Asia and southern China.^1^ Unlike other HSNCC, NPC is radiosensitive and radiotherapy is the primary treatment for nonmetastatic NPC patients.

Post‐radiotherapy evaluation of viable tumor residue is critical to initiate timely salvage treatment and avoid overtreatment. Magnetic resonance imaging (MRI) is well accepted as the mainstay in posttreatment assessment, but distortion of anatomic structures, fibrosis, and inflammation could pose challenges in distinguishing residual tumors from post‐radiotherapy changes.^2^ [18F]‐fluorodeoxyglucose positron emission tomography/computed tomography (FDG‐PET/CT), on the other hand, has incorporated metabolic imaging into morphological imaging. Meta‐analysis suggests that PET/CT enjoyed higher specificity and diagnostic odds ratio in the diagnosis of residual/recurrent NPC compared with MRI, but heterogeneity exists in test characteristics (threshold, comparator, reference standard, timing, etc).^3^, ^4^ Given the potential benefits, Hopkins criteria were introduced into the posttreatment assessment of HSNCC to standardize the interpretation.^5^ Subsequent studies showed a reduction of equivocal results and improved consistency using this scoring system.^6^, ^7^

Limited by small sample sizes of previous studies, whether Hopkins criteria are applicable to NPC is yet to be determined. We conducted this retrospective study to investigate the accuracy of FDG‐PET/CT using Hopkins criteria for the detection of residual NPC. What's more, we explored the effect of different timing on the diagnostic accuracy of the criteria, and if the addition of plasma Epstein‐Barr virus (EBV) DNA level could reduce false positive results.

## 2 MATERIALS AND METHODS

### 2.1 Patients

**Figure 1:**
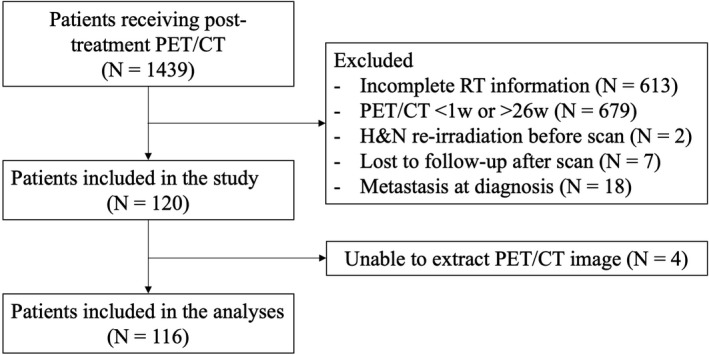
Inclusion and exclusion criteria of the study. RT, radiotherapy; H&N, head and neck

Medical records of 1439 NPC patients who received posttreatment FDG‐PET/CT evaluation at our institution between December 2010 and December 2016 were retrospectively reviewed. Patients with incomplete radiotherapy information, PET/CT scans within 1 week or more than 26 weeks after radiotherapy, H&N re‐irradiation before PET/CT, and metastasis at diagnosis were excluded. Those who were lost to follow‐up soon after PET/CT scan were not evaluable for residual disease and were also excluded. One hundred and twenty patients were included in the study. We failed to extract the PET/CT image of four patients, leading to 116 patients included in the final analyses (Figure 1). PET/CT scans were not mandatory for follow‐up. The scans were ordered at the discretion of physician, usually for patients with regionally advanced disease and/or high baseline EBV levels. Patients were recommended to perform scans 12‐16 weeks after radiotherapy, but early scans may be ordered in cases highly suspicious of residual or metastatic disease. All patients were restaged according to the 8th edition of UICC/AJCC staging system.

### 2.2 FDG‐PET/CT protocol

All PET/CT studies that were performed at our institution used either a Discovery ST16 (two‐dimensional) PET/CT (GE Healthcare) or a Biograph mCT (three‐dimensional) PET/CT (Siemens Healthcare, Henkstr). All patients were scanned from mid‐thigh to skull. The acquisition time per bed position was 3 minutes (two‐dimensional) or 1 minute 30 seconds (three‐dimensional), respectively. Patient with a blood glucose level greater than 11.1 mmol/L at the time of injection was excluded from the study. Patients were injected with 7.4 ± 0.74 MBq (0.2 ± 0.02 mCi) (two‐dimensional), or 3.7 ± 0.37 MBq (0.1 ± 0.01 mCi) of ^18^F‐FDG (three‐dimensional), per kilogram of body weight, respectively. The uptake time was between 50 and 80 minutes. The PET images were reconstructed using ordered‐subsets expectation maximization (two‐dimensional) or tureX plus time‐of‐flight (ultraHD‐PET, three‐dimensional). All PET data were reconstructed with CT‐based attenuation correction. Helical CT images were obtained with a matrix of 500 × 500. Tube voltage was 120 kVp. The current of Discovery ST16 and Biograph mCT were auto modulation (two‐dimensional) and 180 mA (three‐dimensional). Slice thickness was 3.25 mm (two‐dimensional) or 2 mm (three‐dimensional) for PET images, and was 3.75 mm (two‐dimensional) or 3 mm (three‐dimensional) for CT images. Field of view was 50 cm.

PET/CT images and multiplanar reconstructions of PET, CT, or PET/CT were reviewed on a workstation with PET/CT reading software (Advantage Workstation Server; version 2.0, GE Healthcare).

### 2.3 Image interpretation

Cases were scored using the Hopkins criteria^5^. A single circular region of interest (ROI) of 3 cm in diameter was drawn over the right lobe of the liver, avoiding obvious vessels, intrahepatic bile ducts, and metastases. Another single circular ROI of 5 mm in diameter was drawn over the right internal jugular vein (IJV). Scores were obtained for nasopharynx and neck lymph nodes by visual inspection. Focal FDG uptake less than IJV was scored 1. Focal FDG uptake more than IJV but less than liver was scored as 2. Diffuse FDG uptake greater than IJV or liver was scored as 3. Focal FDG uptake more than liver was scored as 4, and intense FDG uptake greater than liver was scored as 5. Scores of 4 or 5 were considered positive for residual tumor. Occasionally, differentiation between scores 1, 2, and 3 by visual observation was difficult. In these cases, the average standardized uptake value of ROIs was taken as the background blood pool SUV reference, and maximum standardized uptake value of lesions was used to assist in visual scoring. All imaging was interpreted by an experienced physician from nuclear medicine department and reviewed by a senior nuclear medicine physician with 7‐year experience. Reviewers were blinded to histological and clinical findings. Disagreement was resolved by discussion.

### 2.4 Plasma EBV DNA

Plasma EBV DNA level was measured by quantitative polymerase chain reaction. Any detectable level of EBV DNA after radiotherapy was considered positive regardless of its copy number.

### 2.5 Follow‐up and outcomes

Follow‐up was recommended at 3‐month intervals for the first 2 years and every 6 months thereafter. Earlier visits might be scheduled based on clinical complaints. Routine examinations included medical history, physical examination, nasopharyngeal endoscopy, MRI with contrast for nasopharynx and neck, chest X‐ray or CT, liver ultrasound or CT, as well as bone scan. Persistent disease was histologically diagnosed by nasopharyngeal biopsy or neck dissection in most cases. Where biopsy was not feasible, persistent disease was clinically confirmed by at least two imaging modalities and patients' response to salvage treatment. Follow‐up time was calculated from the completion of treatment. The overall survival (OS) was measured from the completion of treatment to the date of death from any cause. Disease‐free survival (DFS) was measured from the end of treatment to the date of recurrence, metastasis, or death. Locoregional failure‐free survival (LRFFS) was measured from the completion of treatment to locoregional failure or death.

### 2.6 Statistical analysis

Sensitivity, specificity, accuracy, positive prognostic value (PPV), and negative prognostic value (NPV) were calculated for the entire group, and, respectively, for patients with different metastatic status. As for timing, patients were stratified into three groups: those performed <12 weeks, 12‐16 weeks, and >16 weeks after the completion of treatment. Fisher's exact test was used to compare the diagnostic parameters. Survival curves were calculated by Kaplan‐Meier method, and log‐rank test was used for comparison. Statistical tests were two‐sided and a *P* < .05 was considered significant. SPSS version 21 (IBM) was used for statistical analyses.

## 3 RESULTS

### 3.1 Patient characteristics

A total of 116 patients were included in the analysis. All but one patient received intensity‐modulated radiotherapy (IMRT). Patient characteristics and treatment modalities were summarized in Table 1. Eighty‐seven patients had plasma EBV DNA test at baseline, among whom 73.5% had plasma EBV DNA level >1 × 10^3^. Distant metastases were seen in 50 (43.1%) patients at the time of PET/CT scans. Among the 19 (16.4%) patients diagnosed with residual disease, 15 (78.9%) were confirmed by pathology and four (21.1%) were clinically diagnosed. Ten residual lesions were located in the nasopharynx, 14 lesions were detected in the cervical lymph nodes, and one was clinically diagnosed as residual retropharyngeal lymph node. Among those with residual cervical lymph nodes, 94 patients had EBV DNA tests at the time of PET/CT.

**Table 1:** Patient characteristics (N = 116)

Characteristics	No.	%
Age, y		
Median (range)	43.5 (11‐81)	
Sex		
Male	98	84.5
Female	18	15.5
Histological type		
Keratinizing squamous cell carcinoma	2	1.7
Nonkeratinizing carcinoma	111	95.7
Differentiated	6	
Undifferentiated	105	
Undefined	3	2.6
Tumor stage		
T1 or T2	34	29.3
T3 or T4	82	70.7
Nodal stage		
N0 or N1	40	34.5
N2	47	40.5
N3	29	25.0
Chemotherapy		
Yes	107	92.2
No	9	7.8
RT technique		
IMRT	115	99.1
2D‐RT	1	0.9

Abbreviations: 2DRT, two‐dimensional radiotherapy; IMRT, intensity‐modulated radiotherapy; RT, radiotherapy.

### 3.2 PET accuracy

According to Hopkins criteria, 27 (23.3%) scans were interpreted as positive and 89 (76.7%) scans as negative in the overall assessment. Five scans were false negative, all reading a Hopkins score of 3 for nasopharynx and 2 for neck lymph nodes. Sixty‐six lesions in nasopharynx were scored 3, among which four (6%) were confirmed as positive. Of the patients who were scored 3 in lymph nodes, only one (5%) was found to have residual disease. Overall, the scoring system demonstrated remarkable specificity (86.6%; 95% CI, 78.2%‐92.7%) and NPV (94.4%; 95% CI, 88.7%‐97.3%) as expected. Sensitivity, PPV, and accuracy was 73.7% (95% CI, 48.8%‐90.9%), 51.9% (95% CI, 37.8%‐65.6%), and 84.5% (95% CI, 76.6%‐90.5%), respectively. In patients with residual disease, 10 (62.5%) had detectable plasma EBV DNA. Detectable EBV DNA was not predictive of residual disease (*P* = .272). When EBV DNA was combined with PET/CT results, the overall PPV and accuracy was 50.0% (95% CI, 32.1%‐67.9%) and 83.0% (95% CI, 73.8%‐90.0%), respectively. Accuracies for different locations were summarized in Table 2.

**Table 2:** Diagnostic parameters of Hopkins criteria with or without EBV DNA for different sites

Locations	NPV (%)	PPV (%)	Specificity (%)	Sensitivity (%)	Accuracy (%)
Hopkins criteria^a^					
Nasopharynx	96.1	42.9	92.5	60	89.7
Lymph Nodes	97.7	54.2	88.5	86.7	88.3
Hopkins criteria + EBV DNA^b^					
Nasopharynx	94.2	37.5	94.2	37.5	89.4
Lymph Nodes	93.3	50.0	89.7	61.5	85.7

Abbreviations: NPV, negative predictive value; PPV, positive predictive value; EBV DNA, Epstein‐Barr virus DNA.

a Hopkins score >3 was considered positive for residual tumor.

b Hopkins score >3 in addition to detectable plasma EBV DNA was considered positive for residual tumor.

The median time interval between the date of completion of treatment and PET/CT scans was 14.57 weeks (range, 1‐26 weeks). Twenty‐five (21.6%), 46 (39.7%), and 45 (38.8%) scans were performed <12 weeks, 12‐16 weeks, and >16 weeks after radiotherapy, respectively. Among patients scanned within 12 weeks, 44% of patients received PET/CT at least 2 months after radiotherapy. Diagnostic test characteristics of PET/CT obtained at different posttreatment time intervals were summarized in Table 3. NPV was consistently high across scans performed at different time intervals. PPV for scans acquired <12 weeks, 12‐16 weeks, and >16 weeks was 28.6%, 44.4%, and 72.7%, respectively (*P* = .250). Similar improvement was found in specificity and accuracy, yet the difference was not statistically significant (*P* = .304 for specificity; *P* = .228 for accuracy).

**Table 3:** Diagnostic parameters of Hopkins criteria for different posttreatment time intervals

Time	TN	TP	FN	FP	NPV (%)	PPV (%)	Specificity (%)	Sensitivity (%)	Accuracy (%)
<12 wk	17	2	1	5	94.4	28.6	77.3	66.7	76.0
12‐16 wk	34	4	3	5	91.9	44.4	87.2	57.1	82.6
>16 wk	33	8	1	3	97.1	72.7	91.7	88.9	91.1

Abbreviations: FN, false negative; FP, false positive; NPV, negative predictive value; PPV, positive predictive value; TN, true negative; TP, true positive.

### 3.3 Treatment outcome

**Figure 2:**
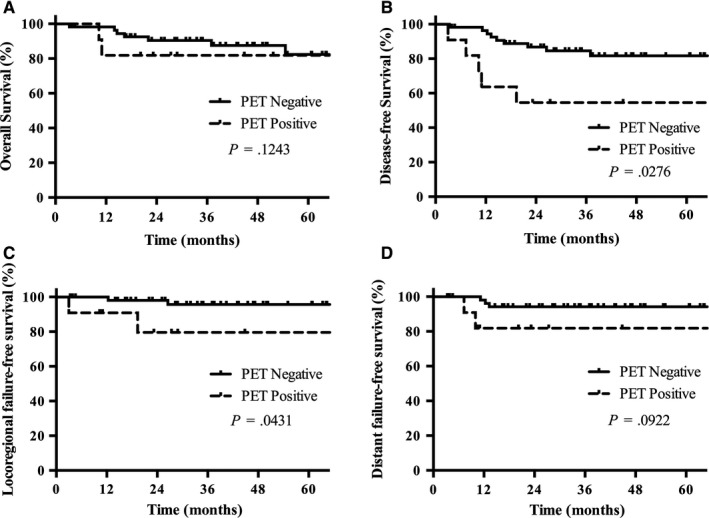
A, Overall survival, B, disease‐free survival, C, locoregional failure‐free survival, and D, distant failure‐free survival of patients with no metastasis at the time of PET/CT scan. PET‐positive was defined as a score of 4 or 5

With a median follow‐up time of 28.3 months (range, 3.3‐92.0 months), the 3‐year OS and DFS for the entire group was 66.5% and 46.6%, respectively. Sixty‐six patients had no metastases at the time of PET/CT scan. The 3‐year OS and DFS in this subgroup was 89.0% and 79.3%, respectively. OS did not differ significantly between the PET‐positive and the PET‐negative group (90.5% vs 81.8%, *P* = .724). The 3‐year LRFFS and 3‐year DFS were significantly higher in PET‐negative group (95.7% vs 79.5%, *P* = .043 for LRFFS; 84.6% vs 54.4%, *P* = .028 for DFS) (Figure 2).

## 4 DISCUSSION

Despite a growing body of evidence to support the application of Hopkins criteria in improving the diagnostic accuracy of therapy response in HSNCC, few data are available for NPC. Our study is distinguished by the inclusion of a relatively large number of NPC patients treated with IMRT and the incorporation of a generally accepted biomarker into the qualitative criteria. Our findings suggest that Hopkins criteria are robust predictive and prognostic indicators in treatment response assessment in NPC patients.

Treatment response evaluation after radiotherapy is crucial for maintaining the balance between timely salvage therapy and overtreatment. Few studies have focused on residual disease, probably for its rarity in the era of IMRT (0.4%‐9%)].^8^, ^9^, ^10^, ^11^ Residual disease could be detected by MRI in 6%‐23% of patients 3 months after IMRT, but 48%‐100% of patients experienced spontaneous remission afterward.^12^, ^13^, ^14^, ^15^ Li et al reported that delayed complete regression on MRI did not jeopardize prognosis, but those who were confirmed residual disease after 9 months had significantly inferior survival.^14^ Thus, imaging relying on anatomic size and morphologic features is not always reliable in the post‐IMRT setting.

As functional and biological imaging, PET/CT has superior diagnostic value in posttherapy assessment of NPC.^3^, ^16^ The diagnostic accuracy of MRI is highly heterogeneous, indicating the underlying challenges in the posttreatment evaluation. Previous studies demonstrated that the sensitivity and specificity of MRI ranged from 15% to 100% and 43% to 90%. For FDG‐PET/CT, the sensitivity was 75%‐100%, and the specificity was 75%‐100%.^3^ Meta‐analyses demonstrated that MRI had an overall sensitivity and specificity of 77% and 76%, while the sensitivity and specificity of FDG‐PET/CT were 90%‐93% and 87%‐93%.^3^, ^4^ The superiority of FDG‐PET/CT is further supported by the data that 12.1% of patients diagnosed with residual disease by conventional imaging could be saved from overtreatment after PET/CT scan.^17^ In addition, posttherapy PET/CT provides more reliable prognostic stratification compared with conventional work‐ups.^18^, ^19^ Despite these advantages, inconsistent image interpretation had led to discordance among different studies.^4^, ^9^, ^10^ The Hopkins 5‐point scale criteria, using IJV and liver as reference sites, have shown exceptional inter‐reader agreement in response assessment in HNSCC. The sensitivity, specificity, NPV, PPV, and accuracy of Hopkins criteria was 65%‐68%, 87%‐92%, 91%‐96%, 33%‐71%, and 85%‐87%, respectively.^5^, ^6^, ^7^ Our study echoes the findings in HSNCC. PPV reported by different studies exhibited remarkable inconsistency, mainly due to a relatively small number of events and various time intervals for posttherapy scanning.

Although posttreatment detectable EBV DNA is a strong indicator for distant metastasis, its association with residual/recurrent disease seems less potent.^20^, ^21^ Some studies suggested its potential correlation with clinical residual disease, but the lack of pathological confirmation limited its clinical significance.^13^, ^22^ Lee et al carried out serial biopsies and plasma EBV DNA tests in NPC patients after IMRT. Only one patient had histologically confirmed residual disease at 12 weeks, while 18 patients had detectable plasma EBV DNA in the absence of residual tumors. Detectable plasma EBV DNA was not associated with locoregional persistence.^11^ Similarly, detectable plasma EBV DNA does not add value to PET/CT in our study. This may be attributed to several reasons. On one hand, some patients in our study had normal or undetectable EBV DNA level at diagnosis. In this subgroup of patients, EBV DNA level did not correspond with the course nor the extent of disease. On the other hand, EBV DNA level undergoes dynamic change after radiotherapy. A large‐scale intelligent platform‐based study showed that 72.4% of patients with detectable plasma EBV DNA within 1 week of therapy experienced spontaneous remission at 3 months.^20^ The time of posttreatment EBV DNA test at PET/CT scans varied in our study, making it extremely difficult to tell whether the addition of EBV DNA could improve the overall accuracy. Comprehensive interpretation of current evidence suggests that for those with detectable EBV DNA after treatment, who may be at higher risk of locoregional persistence/recurrence, functional imaging is required in addition to dynamic surveillance of EBV DNA.

Optimal timing for posttreatment PET/CT scan remains undefined. Kim et al proposed that it was feasible to advance PET/CT scan to 1 month after radiotherapy in HNSCC.^23^ PET/CT performed within 2 months after definitive radiotherapy was significantly less accurate.^24^, ^25^, ^26^ In our study, delayed scan seemed to yield superior PPV. Although this could be partially explained by the reduction of false positive caused by inflammation, higher prevalence of residual disease after 3‐4 months might also contribute to the higher PPV. PPV of posttherapy PET/CT has been considered suboptimal in HNSCC, and delayed scanning proved little difference.^4^, ^27^, ^28^

Our study has several limitations. Firstly, this is a retrospective study and selection bias may exist. PET/CT is not included in the routine follow‐up in our center. Instead, posttreatment PET/CT studies were ordered at clinician's discretion, usually in cases where residual or metastatic disease was suspected. Besides, patients in our study were predominantly locoregionally advanced. Thus, patients included in our study might represent an inferior subgroup, which might explain the above‐average incidence of residual and metastatic disease. Secondly, some residual lesions were not histologically proven where biopsy was not feasible, leading to potential diagnostic errors. Thirdly, inter‐reader agreement has not been examined in our study, as previous studies in HNSCC have already demonstrated excellent inter‐reader reliability.^5^, ^6^, ^7^

## 5 CONCLUSION

Hopkins criteria can effectively rule out residual disease after curative radiotherapy in NPC patients. PPV is suboptimal even when plasma EBV DNA is incorporated. In addition, Hopkins criteria prove to be useful for predicting locoregional failure. Scans should not be performed earlier than 3 months after radiotherapy for low diagnostic accuracy.

## Data Availability

The data that support the findings of this study are available from the corresponding author upon reasonable request.

## References

[1] Chang ET , Adami HO . The enigmatic epidemiology of nasopharyngeal carcinoma. Cancer Epidemiol Biomarkers Prev. 2006;15:1765‐1777. 17035381 10.1158/1055-9965.EPI-06-0353

[2] Ng SH , Liu HM , Ko SF , Hao SP , Chong V . Posttreatment imaging of the nasopharynx. Eur J Radiol. 2002;44:82‐95. 12413677 10.1016/s0720-048x(02)00061-x

[3] Wei J , Pei S , Zhu X . Comparison of (18)F‐FDG PET/CT, MRI and SPECT in the diagnosis of local residual/recurrent nasopharyngeal carcinoma: A meta‐analysis. Oral Oncol. 2016;52:11‐17. 26547126 10.1016/j.oraloncology.2015.10.010

[4] Zhou H , Shen G , Zhang W , Cai H , Zhou Y , Li L . 18F‐FDG PET/CT for the diagnosis of residual or recurrent nasopharyngeal carcinoma after radiotherapy: a metaanalysis. J Nucl Med. 2016;57:342‐347. 26541775 10.2967/jnumed.115.165407

[5] Marcus C , Ciarallo A , Tahari AK , et al. Head and neck PET/CT: therapy response interpretation criteria (Hopkins Criteria)‐interreader reliability, accuracy, and survival outcomes. J Nucl Med. 2014;55:1411‐1416. 24947059 10.2967/jnumed.113.136796 PMC4390037

[6] Kendi AT , Brandon D , Switchenko J , et al. Head and neck PET/CT therapy response interpretation criteria (Hopkins criteria) – external validation study. Am J Nucl Med Mol Imaging. 2017;7:174‐180. 28913156 PMC5596320

[7] Van den Wyngaert T , Helsen N , Carp L , et al. Fluorodeoxyglucose‐positron emission tomography/computed tomography after concurrent chemoradiotherapy in locally advanced head‐and‐neck squamous cell cancer: the ECLYPS study. J Clin Oncol. 2017;35:3458‐3464. 28854069 10.1200/JCO.2017.73.5845

[8] Chan S‐C , Ng S‐H , Chang J‐C , et al. Advantages and pitfalls of 18 F‐fluoro‐2‐deoxy‐D ‐glucose positron emission tomography in detecting locally residual or recurrent nasopharyngeal carcinoma: comparison with magnetic resonance imaging. Eur J Nucl Med Mol Imaging. 2006;33:1032. 16622711 10.1007/s00259-005-0054-6

[9] Chang J , Chan S , Yen T , et al. Differential roles of 18F‐FDG PET in patients with locoregionally advanced nasopharyngeal carcinoma after primary curative therapy: response evaluation and impact on management. J Nucl Med. 2006;66:S431‐S. 16954552

[10] Jeong Y , Jung IH , Kim JS , Chang SK , Lee SW . Clinical significance of the post‐radiotherapy (18)F‐fludeoxyglucose positron emission tomography response in nasopharyngeal cancer. Br J Radiol. 2019;20180045. 10.1259/bjr.20180045 PMC6774585 30102562

[11] Lee V , Kwong D , Leung TW , et al. Post‐radiation plasma Epstein‐Barr virus DNA and local clinical remission after radical intensity‐modulated radiation therapy for nasopharyngeal carcinoma. Clin Oncol. 2016;28:42‐49. 10.1016/j.clon.2015.09.009 26482452

[12] Hong J , Yao Y , Zhang YU , et al. Value of magnetic resonance diffusion‐weighted imaging for the prediction of radiosensitivity in nasopharyngeal carcinoma. Otolaryngol Head Neck Surg. 2013;149:707‐713. 23884282 10.1177/0194599813496537

[13] Lv J‐W , Zhou G‐Q , Li J‐X , et al. Magnetic resonance imaging‐detected tumor residue after intensity‐modulated radiation therapy and its association with post‐radiation plasma Epstein‐Barr virus deoxyribonucleic acid in nasopharyngeal carcinoma. J Cancer. 2017;8:861‐869. 28382149 10.7150/jca.17957 PMC5381175

[14] Li W‐F , Zhang Y , Liu XU , et al. Delayed clinical complete response to intensity‐modulated radiotherapy in nasopharyngeal carcinoma. Oral Oncol. 2017;75:120. 29224808 10.1016/j.oraloncology.2017.10.020

[15] Han F , Xiao W , Wang H , et al. Influence of intensity‐modulated radiotherapy on tumor regression in nasopharyngeal carcinoma. Chin J Radiol Med Pr. 2012;32:204‐206.

[16] Liu T , Xu W , Yan WL , Ye M , Bai YR , Huang G . FDG‐PET, CT, MRI for diagnosis of local residual or recurrent nasopharyngeal carcinoma, which one is the best? A systematic review. Radiother Oncol. 2007;85:327‐335. 18037523 10.1016/j.radonc.2007.11.002

[17] Zheng XK , Chen LH , Wang QS , Wu FB . Influence of [18F] fluorodeoxyglucose positron emission tomography on salvage treatment decision making for locally persistent nasopharyngeal carcinoma. Int J Radiat Oncol Biol Phys. 2006;65:1020‐1025. 16730131 10.1016/j.ijrobp.2006.02.037

[18] Chan SC , Wang HM , Chang TC , et al. Prognostic implications of post‐therapy F‐FDG PET in patients with locoregionally advanced nasopharyngeal carcinoma treated with chemoradiotherapy. Ann Nucl Med. 2013;27:710‐719. 23715831 10.1007/s12149-013-0736-2

[19] Wray R , Sheikhbahaei S , Marcus C , et al. Therapy response assessment and patient outcomes in head and neck squamous cell carcinoma: FDG PET Hopkins criteria versus residual neck node size and morphologic features. Am J Roentgenol. 2016;207:1‐7. 27341273 10.2214/AJR.15.15730

[20] Zhang Y , Tang LL , Li YQ , Liu X , Liu Q , Ma J . Spontaneous remission of residual post‐therapy plasma Epstein‐Barr virus DNA and its prognostic implication in nasopharyngeal carcinoma: a large‐scale, big‐data intelligence platform‐based analysis. Int J Cancer. 2019;144:2313‐2319. 30485420 10.1002/ijc.32021

[21] Lin J‐C , Wang W‐Y , Chen KY , et al. Quantification of plasma Epstein‐Barr virus DNA in patients with advanced nasopharyngeal carcinoma. N Engl J Med. 2004;350:2461‐2470. 15190138 10.1056/NEJMoa032260

[22] Song Y , Xiao H , Yang Z , et al. The predictive value of pre‐ and post‐induction chemotherapy plasma EBV DNA level and tumor volume for the radiosensitivity of locally advanced nasopharyngeal carcinoma. Excli Journal. 2017;16:1268‐1275. 29333129 10.17179/excli2017-752 PMC5763095

[23] Kim SY , Lee SW , Nam SY , et al. The Feasibility of 18F‐FDG PET scans 1 month after completing radiotherapy of squamous cell carcinoma of the head and neck. J Nucl Med. 2007;48:373‐378. 17332614

[24] Greven KM , Williams DW , McGuirt WF , et al. Serial positron emission tomography scans following radiation therapy of patients with head and neck cancer. Head Neck. 2001;23:942‐946. 11754497 10.1002/hed.1136

[25] Leung AS , Rath TJ , Hughes MA , Kim S , Branstetter BF . Optimal timing of first posttreatment FDG PET/CT in head and neck squamous cell carcinoma. Head Neck. 2016;38:E853‐E858. 25917499 10.1002/hed.24112

[26] Nakamura S , Toriihara A , Okochi K , Watanabe H , Shibuya H , Kurabayashi T . Optimal timing of post‐treatment [18F]fluorodeoxyglucose‐PET/CT for patients with head and neck malignancy. Nucl Med Commun. 2013;34:162‐167. 23196675 10.1097/MNM.0b013e32835bdfe3

[27] Gupta T , Master Z , Kannan S , et al. Diagnostic performance of post‐treatment FDG PET or FDG PET/CT imaging in head and neck cancer: a systematic review and meta‐analysis. Eur J Nucl Med Mol Imaging. 2011;38:2083‐2095. 21853309 10.1007/s00259-011-1893-y

[28] Slevin F , Subesinghe M , Ramasamy S , Sen M , Scarsbrook AF , Prestwich RJ . Assessment of outcomes with delayed (18)F‐FDG PET‐CT response assessment in head and neck squamous cell carcinoma. Br J Radiol. 2015;88:20140592. 26081447 10.1259/bjr.20140592 PMC4651393

